# Late Effects of Cancer Treatment, Job Resources, and Burnout Complaints Among Employees With a Breast Cancer Diagnosis 2–10 Years Ago: A Longitudinal Study

**DOI:** 10.3389/fpsyg.2021.793138

**Published:** 2022-01-28

**Authors:** Ingrid G. Boelhouwer, Willemijn Vermeer, Tinka van Vuuren

**Affiliations:** ^1^Department of Organisation, Faculty of Management, Open Universiteit, Heerlen, Netherlands; ^2^Department of Applied Psychology, Amsterdam University of Applied Sciences, Amsterdam, Netherlands; ^3^Loyalis Knowledge & Consult, Heerlen, Netherlands

**Keywords:** burnout complaints, breast cancer, cognitive complaints, employee, fatigue, job resources, late effects

## Abstract

**Purpose:**

The aim of this study was to investigate the effect of possible late effects of cancer treatment (physical complaints, fatigue, and cognitive complaints) and of two job resources (autonomy and supportive leadership style) on future burnout complaints, among employees living 2–10 years beyond breast cancer diagnosis.

**Methods:**

Data at T1 (baseline questionnaire) and at T2 (9 months later) were collected in 2018 and 2019 (*N* = 287). These data were part of a longitudinal study among Dutch speaking workers with a cancer diagnosis 2–10 years ago. All complaints and job resources were self-reported. Longitudinal multivariate regression analyses were executed, controlling for years since diagnosis, living with cancer (recurrence or metastasis), and other chronic or severe diseases. Mediation by baseline burnout complaints was considered.

**Results:**

A higher level of fatigue and cognitive complaints at baseline (T1) resulted in higher future burnout complaints (at T2), with partial mediation by baseline burnout complaints. No effect of physical complaints at T1 was observed. Higher levels of autonomy or a supportive leadership style resulted in lower burnout complaints, with full mediation by baseline burnout complaints. Buffering was observed by autonomy in the relationship of cognitive complaints with future burnout complaints. No moderation was observed by supportive leadership.

**Conclusion:**

The level of burnout complaints among employees 2–10 years beyond breast cancer diagnosis may be an effect of fatigue or cognitive complaints, and awareness of this effect is necessary. Interventions to stimulate supportive leadership and autonomy are advisable, the latter especially in the case of cognitive complaints.

## Introduction

Burnout complaints receive a lot of attention, both in the media, within companies and organizations, among occupational physicians and within the area of work and organizational psychology. The number of research studies into burnout complaints has increased enormously in recent decades (Maslach et al., 2001; Taris et al., 2013). The conceptualization of (clinical) burnout and burnout complaints varies (Demerouti et al., 2021), also depending on the assessment method that even differs between psychological disciplines (van Dam, 2021). However, in the media burnout complaints are often incorrectly referred to as burnout, causing confusion about the intensity and the scope of the phenomenon. It is therefore important to mention that research data show that only a fraction of employees reporting burnout complaints develop a burnout. Nevertheless, in 2018 the percentage of employees in the Netherlands that reported to experience burnout complaints several times a month was 16.4% of the male employees and 18.1% of female employees (RIVM, 2018). Furthermore, data show that 30% of the mental disorders diagnosed by occupational physicians in the Netherlands concerned burnout (Kerncijfers beroepsziekten 2021 | Beroepsziekten.nl, 2021).

To the question how burnout complaints can be prevented or reduced, several answers have been given by means of research based on the well-established Job-Demands Resources (JD-R) model (Schaufeli and Bakker, 2004). Within this model the job-demands are the factors that require effort in the performance of work, possibly resulting in overload. In general, higher demands are associated with burnout (Alarcon, 2011). However, the so-called job resources have a supporting effect, and may reduce burnout complaints and buffer the impact of job demands on burnout complaints (Demerouti et al., 2001; Schaufeli and Bakker, 2004; Bakker et al., 2005, 2014; Xanthopoulou et al., 2007; Bakker and Demerouti, 2017). Two possible job resources are autonomy and a supportive leadership style. In short, autonomy can be described as the possibility to influence one’s work and make decisions, and a supportive leadership style as the degree to which one feels supported by the manager or supervisor. The results of cross-sectional and longitudinal studies indicate that an increase in job demands and a decrease in job resources, predict higher future burnout scores (Schaufeli et al., 2009). Moreover, the two processes are intertwined (Schaufeli and Taris, 2013). In general, it can be said that by balancing job demands and job resources, burnout complaints may be reduced or even prevented.

The World Health Organization recently defined burnout as an occupational phenomenon, characterized by exhaustion, mental distance and decreased personal effectiveness (World Health Organization, 2019). These characteristics are in line with the subscales of the Utrecht Burnout Scale (UBOS) (Schaufeli and van Dierendonck, 2000). Burnout complaints have been studied in relation to various other work-related variables, such as performance (Taris, 2006) or work ability (Ruitenburg et al., 2012). Furthermore, several specific subpopulations within the labor force have been studied, such as nurses (Woo et al., 2020), teachers (Hakanen et al., 2006), or employees with chronic diseases (Boelhouwer et al., 2020). However, workers who have had a cancer diagnosis have not been specifically studied before, while the prevalence of workers living beyond or with cancer is considerable. The prevalence of cancer among the working population in the Netherlands was estimated at a few per cent already more than a decade ago (Kuijpens, 2008). Furthermore, this percentage is expected to continue to rise as a result of the increasing cancer incidence, higher survival rates for several types of cancer, increasing return to work rates and a higher retirement age. As breast cancer is the most common type of cancer at working age, workers beyond cancer diagnosis mostly concern women who were confronted with a breast cancer diagnosis (Ferlay et al., 2021).

Workers beyond cancer diagnosis may experience late effects of cancer treatments, like physical complaints (Ho et al., 2018), fatigue (Prue et al., 2006; Meunier et al., 2007; Reinertsen et al., 2010) or cognitive problems (Wefel et al., 2015). There are cross-sectional research findings showing that these late effects may be associated with impaired occupational functioning, for instance lower work ability, even on the longer term (Boelhouwer et al., 2021b). However, studies on associations of late effects of cancer treatment and burnout complaints are not known to the authors.

It is conceivable that the late effects of cancer treatments aggravate the job demands, as the effort it takes to perform certain tasks may increase. Furthermore, professionals (from human resource management, occupational health care, and physiotherapy) that guide this subpopulation of workers have mentioned that it may be unclear if and how the late effects of cancer treatments and burnout complaints are related to each other (Boelhouwer et al., 2021a). This implies that there is a risk that a burnout diagnosis is established, while the cause of the burnout complaints can be found directly or indirectly in the late effects of the cancer treatments. Therefore, the association of late effects of cancer treatments and burnout complaints should be investigated also quantitatively. Furthermore, the relevance of job resources available in the workplace should be investigated to indicate possible targets for intervention possibly especially important for this population, to start with autonomy and a supportive leadership style in the present study.

Therefore, the aim of this study is to investigate (1) if and to what extent possible late effects of cancer treatments (physical complaints, fatigue, and cognitive complaints) affect the level of future burnout complaints, and (2) to identify a possible direct effect of autonomy or a supportive leadership style on future burnout complaints, as well as buffering of the presumed association of late effects with future burnout complaints, among female salaried employees diagnosed with breast cancer 2–10 years ago.

### Late Effects of Cancer Treatment and Burnout Complaints

Possible late effects of cancer treatments can last for more than decades or even develop many years after treatment (Silver et al., 2013). As previously mentioned, these late effects include among others (1) physical complaints (Ho et al., 2018), (2) fatigue (Prue et al., 2006; Meunier et al., 2007; Reinertsen et al., 2010), and (3) cognitive complaints (Wefel et al., 2015). No quantitative studies are known to the authors about these late effects affecting the level of burnout complaints within the population of workers beyond cancer diagnosis, but data on these problems in relation with burnout complaints among other populations were found.

First, regarding physical problems the available studies tend to be focused on the effect of burnout complaints on physical health, however also the reverse effect has been investigated. A 1-year follow-up study among a sample of Air Force personnel (*N* = 1,009) reported perceived health to predict a decrease in job burnout and the predicted effect of perceived health on job burnout to be significantly larger than the effect of burnout on health (Vinokur et al., 2009). Furthermore, burnout complaints occur more frequently among populations with specific chronic diseases, such as women with musculoskeletal diseases or men with cardiovascular diseases (Honkonen et al., 2006) or women with coronary heart disease (Hallman et al., 2003). However, a cross-sectional study reported that physical chronic diseases (without comorbid mental chronic diseases) were not related to higher burnout complaints (Boelhouwer et al., 2020).

Second, one of the symptoms of burnout is exhaustion (World Health Organization, 2019), which may be similar to extreme fatigue. Several studies have established a relationship between fatigue and burnout complaints, for example a strong positive correlation between compassion fatigue and burnout (Zhang et al., 2018). Acute fatigue was also reported to be more strongly associated with exhaustion than with disengagement among police officers (Basinska et al., 2014). Also, although chronic fatigue syndrome and burnout are reported to have similarities in symptoms, different diagnoses may emerge depending on a perceived cause that may be in the psychological or in the medical direction (Leone et al., 2011).

Third, cognitive functioning is reported to be negatively associated with burnout complaints when cognitive functioning is assessed objectively (by psychometric tests) (Van Der Linden et al., 2005; Deligkaris et al., 2014), as well as subjectively by self-report (Van Dijk et al., 2020). Furthermore, individuals with non-clinical burnout report less cognitive complaints than the clinical burnout patients, but more cognitive problems than healthy controls (Oosterholt et al., 2014).

All in all, a higher level of physical complaints, fatigue or cognitive complaints is expected to have an increasing effect on future burnout complaints beyond the first 2 years after breast cancer diagnosis among employees. Therefore, our first hypothesis (H1) is as follows:

*H1: A higher level of physical complaints, fatigue, or cognitive complaints at T1 (baseline) is associated with higher burnout complaints at T2.*

### Job Resources and Burnout Complaints

Various job resources that are important for the work ability of workers who experience late effects after cancer treatments have emerged in qualitative studies (Boelhouwer et al., 2021a), as well as in cross-sectional quantitative studies focusing on work ability (Boelhouwer et al., 2021b). However, no results with a focus on the impact of job resources on burnout complaints among the working population beyond cancer diagnosis are known to the authors. Nevertheless, because of the relevance of job resources for preventing burnout complaints among other populations, the expectation in the present study is that these results also apply to employees beyond cancer diagnosis. Although any necessary change processes within organizations can take a long time, there are several job resources for which a clear advice can be given in a relatively simple and reasonably practical way in the workplace, namely autonomy and a supportive leadership style.

First, a lack of autonomy is reported to be correlated with burnout risk (Maslach et al., 2001; Kim et al., 2018). Furthermore, a meta-analysis demonstrated negatively associations of autonomy with all three burnout subscales (Alarcon, 2011). Second, leadership style tends to be affected by situational factors and therefore the association of a supportive leadership style with burnout is reported to be complex (Kanste et al., 2007). However, a lack of support from supervisors is regarded as detrimental in relation to burnout complaints, even more so than a lack of support from co-workers (Maslach et al., 2001).

All in all, although research data among workers more than 2 years beyond breast cancer diagnosis are lacking, a higher level of autonomy or a supportive leadership style are expected to be associated with lower burnout complaints among employees 2–10 years beyond breast cancer diagnosis. Hence, our second hypothesis (H2) is as follows:

*H2: Higher levels of autonomy or of supportive leadership style at T1 (baseline) are associated with lower burnout complaints at T2.*

### Buffering by Job Resources of the Association of Late Effects and Burnout Complaints

No quantitative studies among workers beyond cancer diagnosis concerning buffering effects by autonomy or by a supportive leadership style of an association of physical complaints, fatigue or cognitive complaints with burnout complaints are available. However, qualitative studies indicate that these job resources are experienced as relevant factors when workers are confronted with late effects of cancer treatments (Van Maarschalkerweerd et al., 2019). Furthermore, studies among other populations demonstrated the importance of job resources interacting with job demands predicting lower symptoms of burnout (Bakker et al., 2005; Xanthopoulou et al., 2007). Therefore, we expect buffering effects of the above mentioned two job resources in the present study. Hence, our third hypothesis is as follows:

*H3: The relationships between physical complaints, fatigue, or cognitive complaints (late effects) at T1 and burnout complaints at T2 are moderated by autonomy or by a supportive leadership style at T1 such that when the job resource is high, the burnout complaints are lower than when the job resource is low (in other words, we expect buffering by the job resources).*

In addition, three covariates are included in the analyses, namely years since diagnosis, living with cancer (recurrent or metastasis), and the presence of comorbid chronic conditions, as these factors may influence the relationships mentioned because of differences in physical and mental burden. The level of burnout complaints at baseline (T1) will be studied as a possible mediator, as it is expected that burnout complaints will have associations with individual factors as well (Alarcon et al., 2009).

All in all, the present study is quite unique in focusing on burnout complaints among workers beyond cancer diagnosis and the first to investigate the influence of late effects after cancer treatments on future burnout complaints. It is important for professionals to know whether late effects contribute to a possible diagnosis of burnout complaints and whether autonomy or a supportive leadership style require additional attention among workers confronted with late effects of cancer treatment in preventing burnout complaints.

## Materials and Methods

### Procedure

A survey study was carried out (between June 2018 and December 2019) among workers 2 – 10 years beyond cancer diagnosis. Data from workers beyond breast cancer diagnosis and with exclusively salaried employment at T1 (baseline) and at T2 (*N* = 287) were used in the present study. Various methods and channels were used to inform (potential) participants about the study and the first (online) questionnaire such as social media, a short video clip and a website (including the information letter with details regarding storage of the data and confidentiality). An invitation (and a reminder 1 month later) for the second questionnaire was sent out by e-mail to the participants of the first questionnaire. Before the online questionnaire started, the respondent gave informed consent. The Research Ethics Committee (cETO) of the Open Universiteit in the Netherlands assessed the ethical acceptability of the study and agreed with the study design and method (reference cETO: U2018/03891/MQF).

### Measures

The questionnaires included questions concerning demographics and information about health, treatment, and work. For the present study the data to be used were (1) physical complaints, fatigue, cognitive complaints at T1, (2) burnout complaints at T1 and at T2, (3) autonomy and supportive leadership style at T1, and (4) years since diagnosis, living with cancer (recurrence or metastasis), and other chronic or severe diseases at T2.

Physical complaints were measured by a question about complaints caused by ten possible conditions (no, some or many complaints, respectively 0, 1, or 2 points). These conditions were neuropathy, hormonal complaints, hot flushes, bone decalcification, heart complaints (due to treatment), scar adhesions, joint pain, lymph edema, lung problems, and bowel and/or bladder problems. For the level of physical complaints, the total score (0–20) is used. Fatigue was measured by the subjective fatigue subscale (eight items, seven-point Likert scale, 1–7) of the Checklist Individual Strength (CIS) (Vercoulen et al., 1994, 1999). The possible total score for fatigue was 8–56. The Cronbach’s α was 0.91. Cognitive complaints were measured by the Cognitive Failure Questionnaire (CFQ) for subjective cognitive functioning (25 items, five-point Likert scale, 0–4) about the frequency of everyday cognitive errors (Ponds et al., 2006). The possible total score was 0–100. The Cronbach’s α was 0.93. In this study, the score on this scale will be referred to as cognitive complaints.

The general version of the Utrecht Burnout Scale (UBOS-A) was used to measure burnout complaints (Schaufeli and van Dierendonck, 2000). The UBOS-A consists of 15 statements on which the respondent scores on a seven-point scale, ranging from ‘never’ (0) to ‘daily/always’ (6). The items concern complaints on three scales: exhaustion (five items), cynicism or mental distance (four items), and competence (six items). The scores on the latter scale were reversed, so that higher scores correspond to higher burnout complaints. Cronbach’s α were respectively 0.88, 0.84, and 0.83. After administration, the total UBOS-A score is calculated by adding up the scores on all items and indicated as burnout complaints, although only the higher scores will concern clinical complaints.

Autonomy and supportive leadership style, were measured using one of the scales by Van Poppel and Kamphuis (2004). The Cronbach’s α’s at T1 for autonomy (four items, five-point Likert scale) was 0.86, and for supportive leadership style (four items, five-point Likert scale) 0.93.

The year of diagnosis was collected in the first questionnaire, from which the number of years after cancer diagnosis was determined. In the second questionnaire, the respondents indicated whether they were living with cancer (recurrence or metastasis) and whether they had other chronic or severe diseases.

### Participants

The numbers of respondents with a breast cancer diagnosis and exclusively salaried employment at T1 and at T2 was 287.

### Analysis

The data were analyzed using SPSS software, version 25 (IBM Corporation, Armonk NY, United States) for Windows^®^/Apple Mac^®^.

Descriptives were analyzed for the study sample at T2 (*N* = 287) and for the dropouts between T1 and T2 (*N* = 174). Descriptives were demographics (age, gender, and educational level), years since diagnosis (at T1), living with cancer (recurrence or metastasis), and other chronic diseases (at T1 and at T2), late effects (physical complaints, fatigue, cognitive complaints) at T1, burnout complaints (at T1 and at T2), autonomy and supportive leadership style (at T1).

All hypotheses were analyzed by regression analyses, controlling for years since diagnosis, living with cancer (recurrence or metastasis), and other chronic or severe diseases at T2. Missing data were not imputed, and this could result in lower numbers of respondents to be used in analyses. Physical complaints, fatigue, cognitive complaints, autonomy, and supportive leadership style were centered at their means. The hypotheses were tested by regression analyses in six steps; first one step for each of the three covariates, then the late effects together in the fourth step, the two job resources in the fifth step, and the interaction terms to test possible buffering by the job resources of the association of late effects and burnout complaints at T2 in the sixth step. If this regression showed that the late effects significantly predicted burnout complaints at T2, possible mediation by baseline burnout complaints at T1 was analyzed.

To be able to establish if the variable burnout complaints at T1 (baseline) is functioning as a mediator, three assumptions should be met (Baron and Kenny, 1986). First, as already described above, the independent variables should significantly relate to the dependent variable (burnout complaints at T2). Second, the independent variables should significantly relate to the mediator (burnout complaints at T1). Third, when the possible mediator variable (burnout complaints at T1) is added in an additional step of the regression analyses used to verify the first assumption, any significant associations found there are no longer present or reduced (respectively called full mediation or partial mediation by burnout complaints at T1). The testing of these assumptions described above was analyzed and presented in the Tables 3A–C. As described earlier, in these analyzes the interaction terms (to test possible buffering by the job resources) were also included.

**TABLE 1:** Characteristics of participants at T2 (*N* = 287) and dropouts between T1 and T2 (*N* = 174).

Variables at T1	Participants at T2 *N* = 287	Dropouts between T1 and T2 *N* = 174
Age M (*SD*) ^λ^	49.7 (7.72)	48.1 (7.30)
Female gender (N, %)	287 (100%)	174 (100%)
Educational level: Elementary and secondary education Vocational secondary education Higher education Other or missing	77 (27%) 52 (18%) 153 (53%) 5 (2%)	42 (24%) 46 (27%) 84 (48%) 2 (1%)
Years since diagnosis (*M*, *SD*)	4.5 (2.39)	4.6 (2.40)
Living with cancer (recurrence or metastasis) (N, %)	15 (5%)	6 (3%)
Other chronic or severe diseases (N, %)	121 (43%)	67 (39%)
Physical complaints, *M* (*SD*) (0–20)	5.2 (3.82)	5.5 (4.29)
Fatigue, *M* (*SD*) (8–56)	34.1 (12.01)	35.9 (12.14)
Cognitive complaints, *M* (*SD*)^μ^ (0–100)	39.7 (15.71)	43.9 (16.10)
Autonomy, *M* (*SD*) (4–20)	15.2 (3.47)	14.9 (3.78)
Supportive leadership style, *M* (*SD*) (4–20)	13.9 (4.23)	14.4 (4.39)
Burnout complaints score, *M* (*SD*) (15–105)	46.5 (13.95)	47.5 (14.15)
**Variables at T2**	**Participants at T2** ***N* = 287**	
Living with cancer (recurrence or metastasis) (N, %)	17 (6%)	
Other chronic or severe diseases (N, %)	125 (44%)	
Burnout complaints score, M (*SD*) (15–105)	47.1 (13.99)	

*M, mean; SD, standard deviation; N, number of participants.*

*^μ^ Significant difference between participants at T2 and the dropouts (between T1 and T2) at 0.01 level.*

*^λ^ Significant difference between participants at T2 and the dropouts (between T1 and T2) at 0.05 level.*

**TABLE 2:** Correlations of demographics (age, educational level), control variables [years since cancer diagnosis, living with cancer (recurrence or metastasis), other chronic or severe diseases, burnout complaints at T1], dependent variable (burnout complaints at T2), independent variables (physical complaints, fatigue, and cognitive complaints at T1), and possible moderators (autonomy or supportive leadership style at T1) (*N* = 278).

Variables (questionnaire T1 or T2)	1	2	3	4	5	6	7	8	9	10	11	12
(1) Age (T1)	1											
(2) Educational level (T1)	–0.113	1										
(3) Years since cancer diagnosis (T1)	0.212**	0.066	1									
(4) Living with cancer (recurrence or metastasis) (T2)	–0.050	0.001	0.088	1								
(5) Other chronic or severe diseases (T2)	0.133*	−0.126*	–0.029	–0.036	1							
(6) Burnout complaints (T1)	0.003	–0.108	0.003	–0.055	0.196**	1						
(7) Burnout complaints (T2)	–0.023	–0.106	0.029	–0.039	0.250**	0.753**	1					
(8) Physical complaints (T1)	0.101	–0.065	–0.003	–0.001	0.239**	0.282**	0.278**	1				
(9) Fatigue (T1)	–0.050	−0.196**	–0.007	0.006	0.215**	0.513**	0.496**	0.350**	1			
(10) Cognitive complaints (T1)	−0.139*	–0.093	0.020	–0.064	0.143*	0.482**	0.443**	0.369**	0.428**	1		
(11) Autonomy (T1)	0.029	0.135*	–0.029	0.020	–0.042	−0.285**	−0.282**	–0.082	−0.210**	−0.143*	1	
(12) Supportive leadership style (T1)	–0.001	0.011	–0.053	0.043	–0.077	−0.433**	−0.305**	–0.104	−0.215**	−0.188**	0.291**	1

*Correlation is significant at the 0.05 level (two-tailed).

**Correlation is significant at the 0.01 level (two-tailed).

**TABLE 3A:** Multivariate regression analyses for moderation by autonomy or supportive leadership style (at T1) of the association of physical complaints, fatigue, or cognitive complaints (at T1) with future burnout complaints (at T2), controlled by years since diagnosis, living with cancer (recurrence or metastasis) and other chronic or severe diseases.

Step/Variable	*F*	*R* ^2^	Δ *R*^2^	β	β	β	β	β	β
Step 1	0.070	0.000	0.000						
Years since diagnosis at T1				0.017	0.021	0.028	0.028	0.014	0.007
Step 2	0.571	0.005	0.004						
Living with cancer (recurrence or metastasis) at T2					–0.065	–0.064	–0.044	–0.032	–0.007
Step 3	5.622	0.063	0.059						
Other chronic or severe diseases at T2						0.243***	0.132*	0.129*	0.108*
Step 4/late effects at T1	24.376	0.373	0.309						
Physical complaints							0.036	0.032	0.030
Fatigue							0.346***	0.300***	0.297***
Cognitive complaints							0.300***	0.268***	0.292***
Step 5/resources at T1	22.307	0.422	0.050						
Autonomy								−0.107*	−0.119*
Supportive leadership style								−0.179**	−0.216***
Step 6/interaction terms	14.400	0.459	0.036						
Physical complaints × Autonomy									0.208**
Physical complaints × Supportive leadership style									–0.055
Fatigue × Autonomy									–0.025
Fatigue × Supportive leadership style									0.072
Cognitive complaints × Autonomy									−0.165**
Cognitive complaints × Supportive leadership style									–0.042

*Physical complaints, fatigue, cognitive complaints, autonomy, and supportive leadership style were centered at their means.*

*Significant at the < 0.05 level.

**Significant at the < 0.01 level.

***Significant at the < 0.001 level.

**TABLE 3B:** Multivariate regression analyses for moderation by autonomy or supportive leadership style (at T1) of the association of physical complaints, fatigue, or cognitive complaints (at T1) with burnout complaints (at T1), controlled by years since diagnosis, living with cancer (recurrence or metastasis) and other chronic or severe diseases.

Step/Variable	*F*	*R* ^2^	Δ*R*^2^	β	β	β	β	β	β
Step 1	0.225	0.001	0.001						
Years since diagnosis at T1				–0.030	–0.022	–0.017	–0.017	–0.035	–0.043
Step 2	1.803	0.014	0.013						
Living with cancer (recurrence or metastasis) at T2					–0.116	–0.115	–0.094	–0.077	–0.082
Step 3	4.398	0.050	0.036						
Other chronic or severe diseases at T2						0.190**	0.074	0.069	0.074
Step 4/late effects at T1	25.282	0.382	0.332						
Physical complaints							0.046	0.041	0.040
Fatigue							0.351***	0.291***	0.301***
Cognitive complaints							0.314***	0.272***	0.285***
Step 5/resources at T1	26.775	0.467	0.085						
Autonomy								−0.123*	−0.109*
Supportive leadership style								−0.247***	−0.260***
Step 6/interaction terms	15.605	0.479	0.011						
Physical complaints × Autonomy									0.049
Physical complaints × Supportive leadership style									–0.044
Fatigue × Autonomy									–0.039
Fatigue × Supportive leadership style									0.050
Cognitive complaints × Autonomy									–0.076
Cognitive complaints × Supportive leadership style									0.066

*Physical complaints, fatigue, cognitive complaints, autonomy, and supportive leadership style were centered at their means.*

*Significant at the <0.05 level.

**Significant at the <0.01 level.

***Significant at the <0.001 level.

**TABLE 3C:** Multivariate regression analyses for moderation by autonomy or supportive leadership style (at T1) of the association of physical complaints, fatigue, or cognitive complaints (at T1) with future burnout complaints (at T2), controlled by years since diagnosis, living with cancer (recurrence or metastasis), other chronic or severe diseases and burnout complaints at T1.

Step/Variable	*F*	*R* ^2^	Δ*R*^2^	β	β	β	β	β	β	β
Step 1	0.070	0.000	0.000							
Years since diagnosis at T1				0.017	0.021	0.028	0.028	0.014	0.007	0.033
Step 2	0.571	0.005	0.004							
Living with cancer (recurrence or metastasis) at T2					–0.065	–0.064	–0.044	–0.032	–0.007	0.043
Step 3	5.622	0.063	0.059							
Other chronic or severe diseases at T2						0.243***	0.132*	0.129*	0.108*	0.064
Step 4/late effects at T1	24.376	0.373	0.309							
Physical complaints							0.036	0.032	0.030	0.006
Fatigue							0.346***	0.300***	0.297***	0.116*
Cognitive complaints							0.300***	0.260***	0.292***	0.121*
Step 5/resources at T1	22.307	0.422	0.050							
Autonomy								−0.107*	−0.119*	–0.054
Supportive leadership style								−0.179**	−0.216***	–0.060
Step 6/interaction terms	14.400	0.459	0.036							
Physical complaints × Autonomy									0.208**	0.178**
Physical complaints × Supportive leadership style									–0.055	–0.029
Fatigue × Autonomy									–0.025	–0.002
Fatigue × Supportive leadership style									0.072	0.042
Cognitive complaints × Autonomy									−0.165**	−0.119*
Cognitive complaints × Supportive leadership style									–0.042	–0.082
Step 7/mediator										
Burnout complaints at T1	28.952	0.647	0.118							0.601***

*Physical complaints, fatigue, cognitive complaints, autonomy, and supportive leadership style were centered at their means.*

*Significant at the <0.05 level.

**Significant at the <0.01 level.

***Significant at the <0.001 level.

## Results

### Descriptives

At T1 the mean age of the study sample (*N* = 287) was 49.7 years (*SD* 7.72) and the mean number of years since diagnosis was 4.5 years (*SD* 2.39). At T2 6% (*N* = 17) was living with cancer (recurrence or metastasis), and 44% (*N* = 125) had other chronic or severe diseases. Dropouts between T1 and T2 (*N* = 174) were younger and reported a higher level of cognitive complaints. See Table 1.

Burnout complaints at T1 were strongly correlated with burnout complaints at T2 (r = 0.753, *p* < 0.001). A paired sample test resulted in no significant difference between burnout complaints at T1 and burnout complaints at T2. The three late effects showed low mutual correlations, and the same was observed for the job resources. The late effects were correlated positively (*p* < 0.01) both with burnout complaints at T1, and with burnout complaints at T2. The two job resources were correlated negatively (*p* < 0.01) both with burnout complaints at T1, and with burnout complaints at T2. See Table 2.

### Hypothesis Testing

The stepwise multivariate regression analyses excluded cases with missing values and resulted in *N* = 253. The final model explained 46% of the observed variance.

One of the three covariates showed a significant association with burnout complaints at T2, namely other chronic or severe diseases (β = 0.108, *p* < 0.05). Years since diagnosis and living with cancer (recurrence or metastasis) and were not associated with burnout complaints at T2. See Table 3A.

Thereafter hypothesis H1 was tested in step 4 of the multivariate regression analyses, with physical complaints, fatigue, and cognitive complaints at T1 as possible predictors of burnout complaints at T2. A higher degree of the number of physical complaints did not predict higher burnout complaints at T2 (β = 0.030, *p* = 0.582), however, a higher level of fatigue and a higher level of cognitive complaints did (respectively β = 0.297, *p* < 0.001 and β = 0.292, *p* < 0.001). See Table 3A. Additionally, mediation analyses were executed. The results showed partial mediation by burnout complaints at T1 in the relationship of fatigue and cognitive complaints with burnout complaints at T2. This can be deduced from the fact that all three assumptions for mediation were met for fatigue and for cognitive complaints, as these variables showed significant associations with the dependent variables in all required analyses. In the additional step in the regression analysis (introducing burnout complaints at T1 as a possible mediator), the associations were β = 0.116, *p* < 0.05 for fatigue and β = 0.121, *p* < 0.05 for cognitive complaints. As the associations were significant to a lesser extent, this means the mediation by burnout complaints at T1 was partial. See Tables 3A–C.

Hypothesis H2 was tested in step 5 of the above-described multivariate regression analyses, with the job resources (autonomy and supportive leadership style) at T1 as possible predictors, and burnout complaints at T2 as dependent variable. Our hypothesis was confirmed. A higher level of autonomy or a supportive leadership style predicted lower burnout complaints at T2 (respectively β = –0.119, *p* < 0.05 and β = –0.216, *p* < 0.001). See Table 3A. The results showed full mediation by burnout complaints at T1 in the relationship of the job resources with burnout complaints at T2. This can be deduced from the fact that all three assumptions for mediation were met. In the additional step in the regression analysis (introducing burnout complaints at T1 as a possible mediator), the associations were β = –0.054, *p* = 0.223 for autonomy and β = –0.060, *p* = 0.179 for supportive leadership style. As the associations were not significant anymore, this means burnout complaints at T1 fully mediated the association between the job resources and burnout complaints at T2. See Tables 3A–C.

Hypotheses H3 concerned the expected buffering of the job resources (autonomy and supportive leadership style) at T1 of the relationships between physical complaints, fatigue, or cognitive complaints at T1 and burnout complaints at T2. Buffering was observed by autonomy of the association of cognitive complaints at T1 with burnout complaints at T2 (β = –0.165, *p* < 0.01). Furthermore, there was one other case of moderation (no buffering, but a worsening) by autonomy of the association of physical complaints with burnout complaints at T2 (β = 0.208, *p* < 0.01). In the analysis of H3, no mediation by burnout complaints at T1 was observed as the regression analysis with burnout complaints at T1 as dependent variable did not show any significant result for the interaction terms. See Tables 3A–C.

## Discussion

The result that higher levels of fatigue and cognitive complaints cause higher future burnout complaints (measured with the UBOS-A, a validated measurement tool) among a population of employees with a breast cancer diagnosis 2–10 years ago is remarkable. Burnout complaints appeared to be quite stable in time, and further analyses showed that baseline burnout complaints partially mediated the relation between fatigue and cognitive complaints at T1, and burnout complaints at T2. This causal relationship between fatigue and cognitive complaints on one side and future burnout complaints on the other side among employees more than 2 years beyond breast cancer diagnosis is not reported before. Studies among other populations did report negative cross-sectional associations of self-reported fatigue (Zhang et al., 2018) or of cognitive complaints (Van Dijk et al., 2020) with self-reported burnout complaints. Furthermore, also cross-sectional associations between cognitive complaints and fatigue have been reported among cancer survivors in several other studies (Todd et al., 2011; Dorland et al., 2016), as well in the present study (see Table 2). However, no comparisons can be made with other longitudinal studies among workers more than 2 years beyond cancer diagnosis as these are not available.

It was already mentioned that it may be difficult to distinguish burnout complaints from the late effects as a result of cancer treatments (Boelhouwer et al., 2021a). This possibility was also brought forward in a focus group study among cancer survivors and professionals (Klaver et al., 2020). Also, a cross-sectional association between fatigue or cognitive late effects and burnout complaints may increase the risk to mistakenly interpret a late effect of cancer treatment as an aspect of burn-out symptomology. In any case, a correct diagnosis among workers confronted with late effects of cancer treatments requires an examination by a professional with the appropriate background who is allowed to make such a diagnosis and, not just by filling in a questionnaire, even if this is a validated measuring instrument. Furthermore, as self-reported cognitive complaints do not have to go together with objectively established complaints (Poppelreuter et al., 2004), it can also be a consideration to perform a neuropsychological examination.

As expected, the level of autonomy and supportive leadership style did predict lower future burnout complaints in the present longitudinal study, however, this effect changed into non-significant when controlled for burnout complaints at baseline, as burnout complaints at baseline have a fully mediating effect. Cross-sectional studies among this population also reported positive associations with burnout complaints for these job resources (Kanste et al., 2007; Alarcon, 2011; Kim et al., 2018). These results can be explained by the high degree of stability of the burnout symptoms. Buffering was observed by autonomy in the relation of cognitive complaints and higher future burnout complaints, if controlled for burnout complaints at baseline. As no research into interventions for burnout complaints among employees more than 2 years beyond cancer diagnosis are known, this is an interesting finding.

Physical complaints did not affect the level of burnout complaints in the current analyses, which is unexpected as we took note of some other studies where this was the case (Hallman et al., 2003; Honkonen et al., 2006; Vinokur et al., 2009). Against expectations a negative moderation caused by autonomy in the case of physical complaints was found. This result indicates that more autonomy may worsen burnout complaints with an increase of physical complaints, so it may be necessary to carefully monitor how much autonomy is desirable and favorable in the case of more physical complaints. It is possible that workers with many physical late effects receive so much support that at the same time this reduces their autonomy or maybe more autonomy in the case of physical complaints increases work demands even more. In short, in a situation in which the employee has many physical complaints, it is necessary to consciously adjust how much autonomy is experienced as positive by the employee.

Of course, we have to take into account that this study result may be influenced by certain limitations in the design of this study. An important limitation is the possibility that the experienced complaints are not (completely) caused by the cancer treatments, but that other causes also play a role. Only in studies with very large cohorts that are followed for many years is it possible to also make measurements before an occurring cancer diagnoses and to compare these measurements on the same factors in the years after the cancer diagnosis and treatment. Furthermore, hindering late effects may cause stress. Elevated levels of stress affecting burnout measures are reported among individuals experiencing short-term stress (van Dam, 2021). Moreover, all data were self-reported and this may have led to various sources of common method bias (Podsakoff et al., 2003). However, existing, validated scales were used as much as possible. In this regard, it is important to note that burnout complaints were assessed by the UBOS-A, while more recently a new assessment tool, the Burnout Assessment Tool (BAT), is also available. The BAT is based on other core dimensions, namely exhaustion, mental distance, and impaired emotional and cognitive impairment (Schaufeli et al., 2020a,b). It could be that the use of the BAT makes it even more urgent to be aware of the possibility of cognitive complaints in workers who have undergone cancer treatments in the past. Furthermore, cognitive complaints in the present study were not assessed by neuropsychological testing, while results on these objective data and self-reported, subjective data are known to show little correlation (Poppelreuter et al., 2004). Moreover, the results on a self-report scale may be associated with psychosocial factors (Pullens et al., 2010; Feuerstein, 2011; Boscher et al., 2020). Also, the dropouts between the two questionnaires had significantly more cognitive complaints at baseline, and a healthy worker effect may have influenced the results, with not exactly known effects. Finally, it is important to emphasize we cannot report about the occurrence of burnout complaints among this population in general.

To summarize, the present study indicates that fatigue and cognitive complaints increase future burnout complaints among employees 2–10 years beyond breast cancer diagnosis, which is a novel finding. Furthermore, a supportive leadership style and autonomy are regarded as important, the latter especially if cognitive complaints are at higher levels. It is important to also investigate other job resources (like job crafting of coaching) for their positive effect among these workers. In other words, it is very important to conduct more studies into effective job resources specifically to protect workers beyond cancer diagnosis at risk of higher burnout complaints in the future.

Finally, it is important for managers and human resource management within companies and organizations and for other professionals guiding these employees, to realize which late effects of cancer treatments may present an extra risk to show and develop higher levels of the self-reported burnout complaints and to know what can be done in the workplace to reduce or even prevent increasing burnout complaints. Therefore, it is important to always be alert if it is known that an employee has been treated for cancer and therefore possibly experiences late effects of the treatments, in order to include this information in the choice of interventions. Possibly, for employees who are known to have undergone cancer treatments, an additional focus on the treatment of possible fatigue or cognitive complaints from the psycho-oncological perspective may be considered, apart from the attention for factors in the work situation.

## Data Availability Statement

The original contributions presented in the study are included in the article, further inquiries can be directed to the corresponding author/s.

## Ethics Statement

The studies involving human participants were reviewed and approved by the Research Ethics Committee (cETO) of the Open Universiteit in the Netherlands who assessed the ethical acceptability of the study and agreed with the study design and method (reference cETO: U2018/03891/MQF). The participants provided their written informed consent to participate in this study.

## Author Contributions

IB, WV, and TV developed the study design. IB was responsible for the data collection. IB prepared the data analysis with support from TV and WV. IB wrote the first draft of the manuscript and the later drafts of the manuscript were adjusted by all authors in collaboration. All the authors read and approved the submitted version.

## Conflict of Interest

TV was employed by the company Loyalis Knowledge & Consult. The remaining authors declare that the research was conducted in the absence of any commercial or financial relationships that could be construed as a potential conflict of interest.

## Publisher’s Note

All claims expressed in this article are solely those of the authors and do not necessarily represent those of their affiliated organizations, or those of the publisher, the editors and the reviewers. Any product that may be evaluated in this article, or claim that may be made by its manufacturer, is not guaranteed or endorsed by the publisher.
